# Efficacy and Safety of Non‐Cross‐Linked Hyaluronic Acid Mesotherapy for Post‐Acne Erythema: A Split‐Face, Prospective, Randomized, Single‐Center Trial

**DOI:** 10.1111/jocd.70813

**Published:** 2026-05-05

**Authors:** Di Zhang, Yuan Chang, Yue Bai, Ansheng Tan, Zixuan He, Yu Han, Anxin Chen, Fenglin Zhuo

**Affiliations:** ^1^ Department of Dermatology Capital Medical University, Beijing Friendship Hospital Beijing China

**Keywords:** mesotherapy, non‐cross‐linked hyaluronic acid, post‐acne erythema, randomized controlled trial

## Abstract

**Background:**

Post‐acne erythema (PAE) is one of the most common inflammatory sequelae of acne. Although various therapeutic approaches are currently available, many are limited by suboptimal efficacy, poor tolerability, or high costs. This trial aims to evaluate the clinical efficacy and safety of non‐cross‐linked hyaluronic acid (HA) mesotherapy for the treatment of PAE.

**Methods:**

A total of 25 patients aged 18–35 years with clinically diagnosed PAE and bilaterally symmetrical facial lesions were enrolled. Three treatment sessions were performed at 4‐week intervals. The primary outcome was the Clinician Erythema Assessment Scale (CEAS) score assessed at baseline and week 12. Secondary endpoints included the Dermatology Life Quality Index (DLQI), Patient Self‐Assessment (PSA), VISIA skin analysis (red areas, pores, texture), CK measurement parameters (erythema index, melanin index, skin hydration, transepidermal water loss), dermoscopic imaging, adverse effects, and relapse.

**Results:**

Finally, 23 patients completed all treatment sessions. By week 12, the mesotherapy group showed significantly lower CEAS scores compared to the control group. In addition, patients in the mesotherapy group reported higher satisfaction levels. Improvements in red areas, pores, texture, erythema index, skin hydration, transepidermal water loss, and dermoscopic imaging were also more marked in the mesotherapy group. There was a significant change in the DLQI score, too. No serious adverse events or erythema flares occurred during the study. Among the 23 patients who completed the trial, only one experienced acne relapse.

**Conclusion:**

According to the results of this study, non‐cross‐linked HA mesotherapy is a promising and useful therapeutic option for PAE.

## 1 Introduction

Acne is a common chronic inflammatory skin disorder primarily of the pilosebaceous units [1], affecting up to 85% of adolescents globally [2]. Although primary acne lesions often resolve with treatment [3], many patients may develop clinical sequelae following the resolution of inflammatory papules or pustules. These include acne scars (atrophic, hypertrophic, or keloidal), post‐inflammatory hyperpigmentation (PIH), and post‐acne erythema (PAE). Among these, PAE is one of the most frequent sequelae, particularly in individuals with lighter skin types [1].

PAE is a consequence of cutaneous inflammation. Although its exact pathogenesis remains unclear, its clinical manifestations include persistent erythematous macules and telangiectasia [2]. The thinning of the epidermis after the resolution of acne lesions may exacerbate the appearance of erythema. Some evidence also suggests that PAE may be related to stratum corneum impairment and insufficient skin hydration [4]. Some of these lesions may resolve spontaneously, but persistent PAE often lasts for several years without interventional treatment in most patients. This is cosmetically unacceptable and often leads to significant psychological, social, and financial burdens for affected patients [3].

Conventional depigmenting or anti‐inflammatory agents often fail to provide satisfactory results in treating PAE, as this condition is essentially a vascular rather than a pigmentary disorder. Current treatment options include topical vasoconstrictors (e.g., brimonidine [5] and oxymetazoline [6]), energy‐based devices (e.g., pulsed dye laser (PDL) [7, 8] and intense pulsed light (IPL) [2, 3]), chemical peels (e.g., azelaic acid [9]), and skin lightening agents (such as vitamin C [10] and tranexamic acid [11]). However, these approaches may be limited by suboptimal efficacy, poor tolerability, or high treatment costs. Therefore, there is an urgent need to explore a safe, effective, and well‐tolerated treatment approach for PAE.

Hyaluronic acid (HA), a naturally occurring linear polysaccharide in the extracellular matrix (ECM), is well known for its excellent moisturization capacity, biocompatibility, and tissue‐repairing properties [12]. In recent years, non‐cross‐linked HA has been widely used in skin rejuvenation and barrier restoration therapies [13, 14]. Compared to cross‐linked HA, non‐cross‐linked HA has a lower molecular weight, more uniform distribution, and stronger hydrophilicity. Studies have shown that HA can regulate local inflammatory responses by binding to Toll‐like receptor 4 (TLR4) on the surface of keratinocytes and immune cells. This interaction suppresses the release of pro‐inflammatory cytokines such as TNF‐α and IL‐6, thereby inhibiting the pro‐inflammatory effects of the DAMP/TLR4 signaling pathway [15].

To date, no studies have specifically evaluated the efficacy of non‐cross‐linked HA mesotherapy in the treatment of PAE. The study aimed to assess the clinical efficacy and safety of non‐cross‐linked HA mesotherapy for the treatment of PAE.

## 2 Materials and Methods

### 2.1 Methods

A split‐face, prospective, randomized, single‐center trial was conducted in the Department of Dermatology at Beijing Friendship Hospital. From October 2023 to October 2024, a total of 25 patients with PAE were enrolled through outpatient clinics and online recruitment platforms. Written informed consent was obtained from each participant prior to study enrollment. This study protocol complies with the Declaration of Helsinki and all applicable amendments, and was approved by the Ethics Review Committee of Beijing Friendship Hospital (2023‐P2‐209‐02). The clinical trial registry number of this study is ChiCTR2300075561 (https://www.chictr.org.cn/showproj.html?proj=203400).

### 2.2 Patient Selection

Inclusion criteria included: patients aged 18–35 years with clinically confirmed PAE and bilateral symmetrical facial lesions. Exclusion criteria included: presence of active acne; history of keloid formation or hypertrophic scarring; bleeding disorders or current use of anticoagulation medications; active skin infections or inflammatory dermatoses; severe chronic systemic diseases; pregnancy or lactation; allergy to components of the study drug; having received facial aesthetic treatments using fillers or energy‐based devices within 3 months prior to enrollment. In addition, participants agreed to avoid any cosmetic or medical treatments on the face throughout the study period.

### 2.3 Randomization

Random numbers were generated in advance using a random number table and made into numbered slips, which were placed in an opaque box. Each participant drew one slip. If the number was odd, the left side was used as the mesotherapy group. If the number was even, the right side was used as the mesotherapy group.

### 2.4 Treatment Protocols

The study consisted of three injection sessions at four‐week intervals. At baseline, patient information was collected, including age, gender, height, weight, age of acne onset, duration of acne, duration of post‐acne erythema, and Fitzpatrick skin type. For the mesotherapy side, to minimize pain, topical anesthesia was performed by applying 5% compound lidocaine cream for 30 min. After removing the cream, the mesotherapy side was disinfected once with povidone‐iodine and subsequently deiodinated with 75% alcohol. Mesotherapy (Hi Body 2.5, Beijing Aimeike Technology Development Co. Ltd., China) injections were then performed, using an automatic and rapid injection mode, with a negative pressure of 20% and a fixed dose of 0.0312 mL per injection point. For areas without erythema, the needle length was set at 0.5–1.0 mm. For erythematous areas, the length was increased to 1.0–2.0 mm to induce pinpoint bleeding and ensure an adequate treatment response. At last, a cooling facial mask was applied for 20 min. Participants were instructed to avoid water contact for 24 h after treatment, apply one facial mask daily for the following three days, and use a moisturizer (Cetaphil, Galderma, France) twice daily with strict photoprotection throughout the study period.

### 2.5 Efficacy Assessment

#### 2.5.1 Instrument Assessment

At baseline and week 12 (4 weeks after the final treatment session), prior to performing facial imaging and skin measurements, participants were asked to cleanse their faces and rest for 30 min in a constant temperature environment (24 ℃, 50% humidity). Subsequently, facial imaging was taken by the VISIA system (Canfield Scientific Inc., New York, United States) to assess facial redness, texture, and pore condition. After that, we selected the midpoint of the line connecting the left tragus and the lowest point of the left ala nasi (similarly on the right side) as the measurement site. Skin hydration, transepidermal water loss (TEWL), melanin index, and erythema index were tested using the CK multiple‐probe adapter (MPA580, Courage and Khazaka Electronic GmbH, Germany). Measurements were performed in triplicate on each side of the face to obtain the average value. The same sites and consistent pressure were applied across all measurements. Finally, a dermatoscope at 40× magnification (Hongxin Medical Equipment Co. Ltd., Beijing, China) was used to photograph two representative lesions per side. The same lesions were used at each time point to assess improvements in color, area, and degree of telangiectasia before and after treatment.

#### 2.5.2 Physician's Assessment

At baseline and week 12, the severity of PAE was graded by three dermatologists who did not participate in the treatment, based on left and right facial images taken by VISIA skin analysis. At each time point, the final score for each side of the face was calculated as the mean of the scores given by three evaluators (rounded to one decimal place). Erythema improvements were evaluated using the Clinician Erythema Assessment Scale (CEAS), as shown in Table 1.

**TABLE 1:** Clinician Erythema Assessment Scale.

Score	Degree	Description
0	Clear	Clear skin with no signs of erythema
1	Almost clear	Slight redness
2	Mild	Definite redness
3	Moderate	Marked redness
4	Severe	Fiery redness

#### 2.5.3 Patient's Assessment

The Dermatology Life Quality Index (DLQI) questionnaire was assessed in patients at baseline and week 12. It consists of 10 questions with a 0–3 score for each answer. The total DLQI score was calculated by summing the scores of all 10 questions, with a score range of 0 to 30. The higher the score, the greater the impairment in quality of life. Each participant was asked to assess the therapeutic effect on both sides of the face at week 12, using the Patient Self‐Assessment (PSA) scale, as follows: 0 = no improvement in erythema, 1 = 1%–24% improvement, 2 = 25%–49% improvement, 3 = 50%–74% improvement, 4 = 75%–100% improvement.

#### 2.5.4 Safety Assessment

All adverse effects occurring at baseline and throughout the study were recorded by questioning and examining the participants.

#### 2.5.5 Relapse

At week 24, four months after the final treatment, patients who completed treatment were followed up to evaluate acne relapse. Relapse was defined as the presence of more than 15 open or closed comedones, or a combination of both, or more than 15 papulopustular lesions, or two or more nodulocystic lesions [16].

The primary endpoint of this study was to assess improvements in the CEAS score at baseline and week 12. The secondary endpoints included DLQI, PSA, VISIA skin analysis, CK measurement parameters, dermoscopic imaging, adverse effects, and relapse rate in two groups.

### 2.6 Statistical Analysis

Statistical analyses were performed using the SPSS 27.0 (IBM Corp., Armonk, NY, USA). Paired *t*‐tests were used when the data were normally distributed, and the Wilcoxon matched‐pairs signed‐rank test was employed when the data were not normally distributed. *p* < 0.05 was considered statistically significant. For the data described in the tables, normally distributed data were shown as mean ± SEM, and median with range was used for non‐normally distributed data.

## 3 Results

### 3.1 Patient Demographics

**FIGURE 1:**
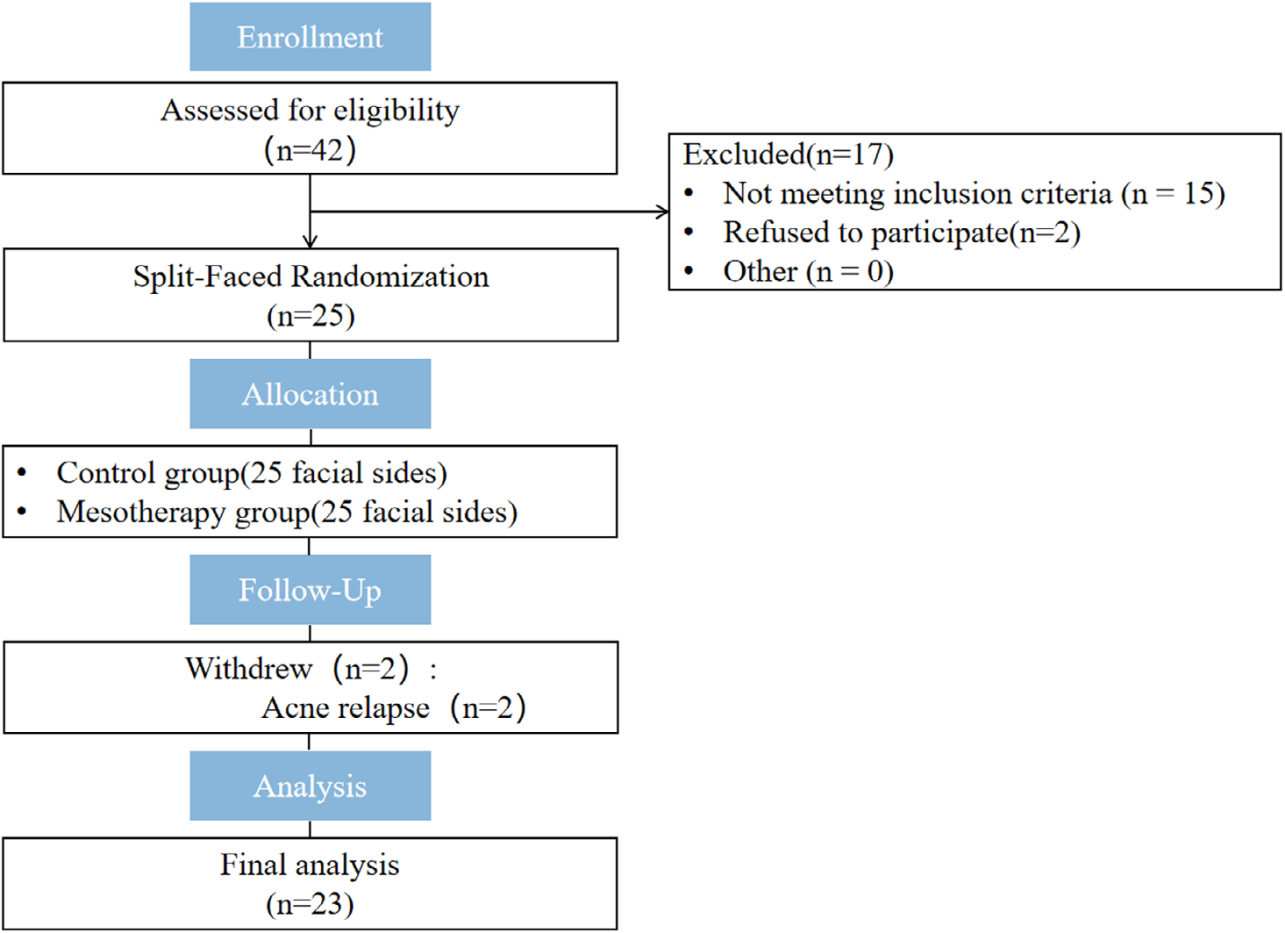
Flow chart of the study.

Out of 25 patients, 23 completed the study, and 2 dropped out during treatment sessions due to acne relapse. The flowchart of the study is shown in Figure 1. Among the participants, nineteen were female and four were male. They were aged 18–30 years, with a mean age of 24.0 ± 3.3 years and a disease duration of 1–6 months (mean ± SD: 3.3 ± 1.4 months). Regarding the age of onset, most patients (13 subjects, 56.52%) were aged 22–25 years; only three patients (13.04%) were aged 18–21 years. Most patients were categorized as Fitzpatrick skin type IV (12 patients, 52.17%). The median duration of acne was 36 months, with a range of 3 to 108 months. Patient demographics are summarized in Table 2.

**TABLE 2:** Demographic and baseline characteristics of patients.

Characteristics	Data
Gender	
Male	4
Female	19
Age (years)	
Mean ± SD	24.0 ± 3.3
Age on set (years)	
18–21	3
22–25	13
26–30	7
BMI (kg/m^2^)	
Mean ± SD	20.7 ± 2.9
Duration of acne (months)	
Median (IQR)	36 (6–84)
Duration of PAE (months)	
Mean ± SD	3.3 ± 1.4
Fitzpatrick skin type	
II	1
III	4
IV	12
V	6

### 3.2 Clinical Efficacy

#### 3.2.1 Clinician Erythema Assessment Scale

**FIGURE 2:**
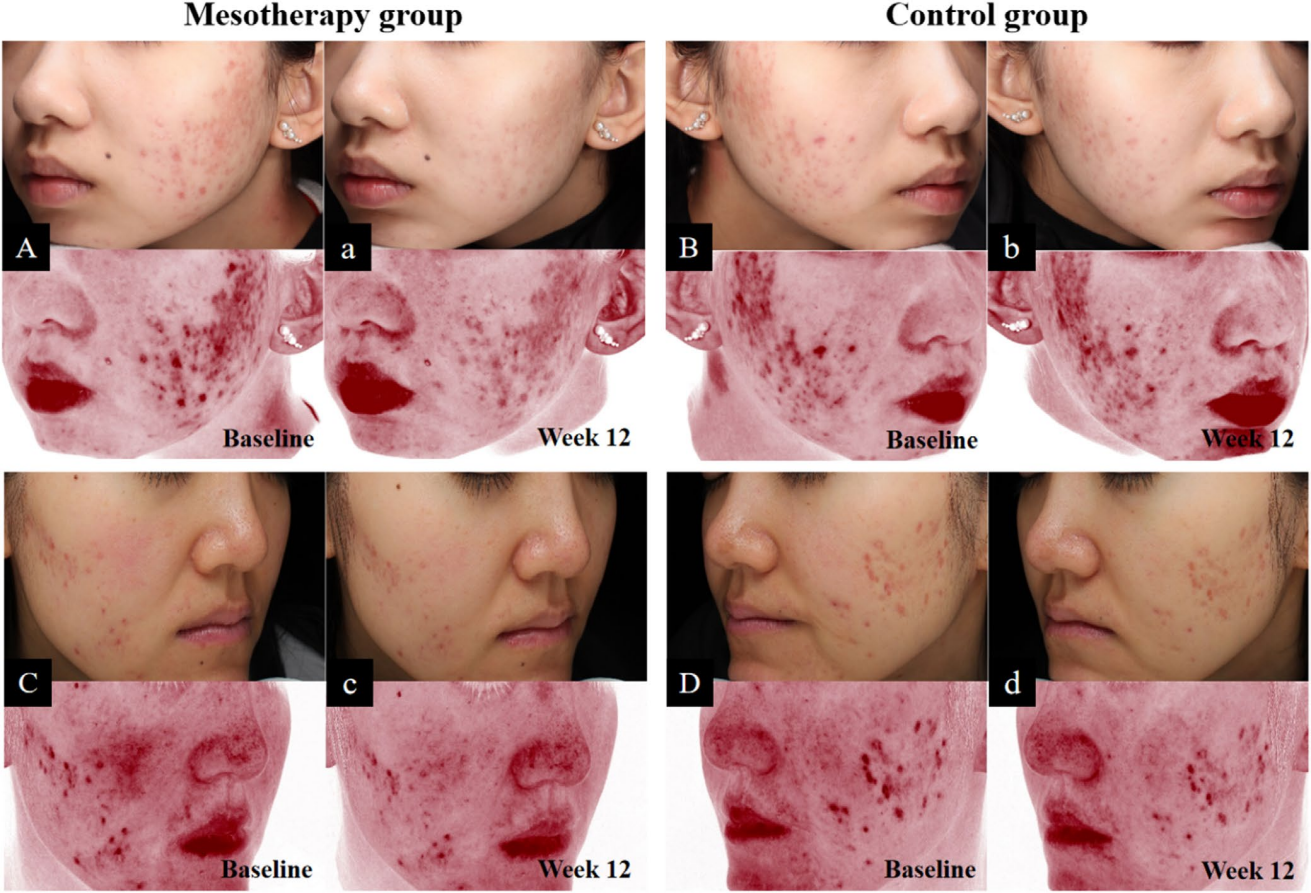
Comparison of clinical images (top) and VISIA images of the red areas (bottom) at baseline and at week 12. (A–D) Baseline. (a–d) Week 12. (A, a, C, c) Mesotherapy group. (B, b, D, d) Control group.

Before treatment, there were no significant differences in CEAS scores between the two groups (*p* = 0.675). Compared to baseline, CEAS scores decreased in both groups. However, only the mesotherapy group showed a statistically significant change (*p* < 0.001). Moreover, the score in the mesotherapy group was significantly lower than that in the control group (*p* = 0.042), as shown in Figure 2 and Table 3.

**TABLE 3:** Comparison of CEAS between the control group and the mesotherapy group.

	Baseline	Week 12	*p*
Control group	2.8 ± 0.8	2.3 ± 0.9	0.126
Mesotherapy group	2.8 ± 0.6	1.8 ± 0.6	< 0.001*
*p*	0.675	0.042*	

* *p* < 0.05.

#### 3.2.2 Patient Assessment

**FIGURE 3:**
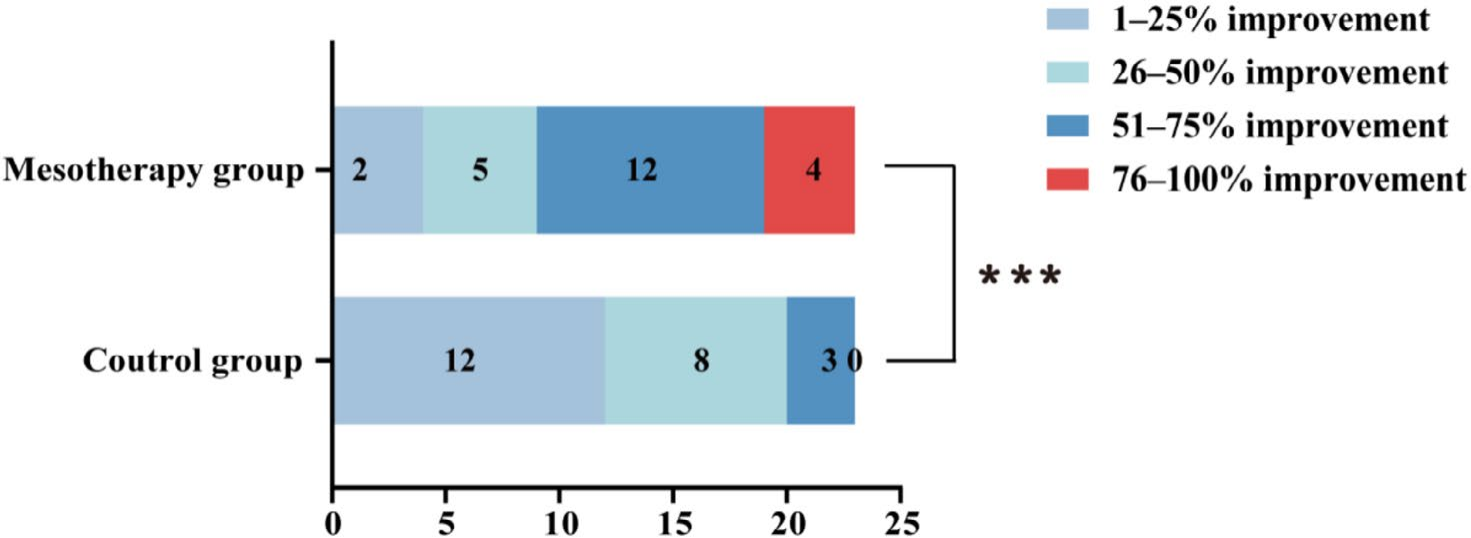
Comparison of the distribution of patient self‐assessment between control group and mesotherapy group at week 12. ***p < 0.001.

A significantly greater proportion of patients showed better clinical improvement in the mesotherapy group (*p* < 0.001, Figure 3). At week 12, in the control group, the DLQI score changed from 13.6 ± 3.3 at baseline to 9.3 ± 3.1, with a statistically significant difference (*p* < 0.001).

#### 3.2.3 VISIA Skin Analysis

**FIGURE 4:**
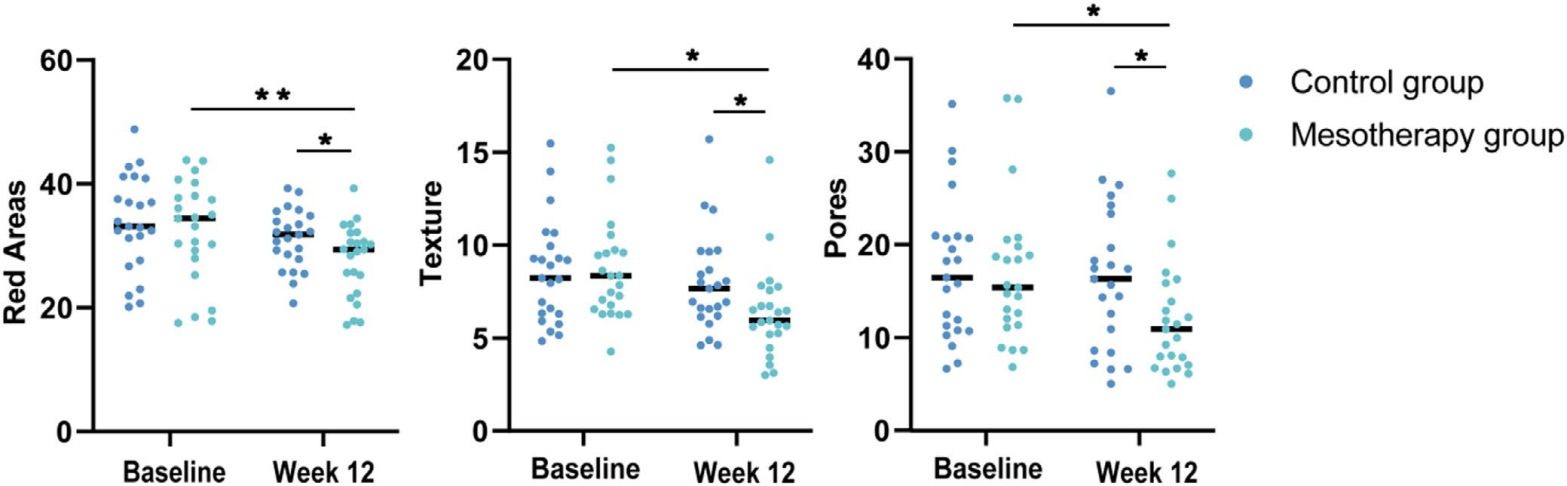
Comparison graphs of parameters measured by VISIA. *p < 0.05, **p < 0.01*.

There were no significant differences between the two groups in red area, texture, and pore scores at baseline (*p* > 0.05). At week 12, all scores decreased in the mesotherapy group compared to baseline (*p* < 0.05) while no changes were observed in the control group. Consequently, all scores in the mesotherapy group were significantly lower than those in the control group (*p* < 0.05). Representative data are shown in Figure 4 and Table 4.

**TABLE 4:** Comparison of parameters measured by the VISIA system.

	Baseline	Week 12	*p*
Red areas			
Control group	33.67 ± 7.75	31.12 ± 4.77	0.188
Mesotherapy group	32.36 ± 8.21	27.69 ± 5.89	0.005*
*p*‐value	0.581	0.035*	
Texture			
Control group	8.55 ± 2.80	7.98 ± 2.64	0.207
Mesotherapy group	8.75 ± 2.79	6.37 ± 2.49	0.010*
*p*‐value	0.767	0.013*	
Pores			
Control group	17.30 ± 7.60	16.53 ± 8.01	0.153
Mesotherapy group	16.89 ± 7.74	12.00 ± 6.03	0.018*
*p*	0.784	0.040*	

* *p* < 0.05.

#### 3.2.4 CK Tests

**FIGURE 5:**
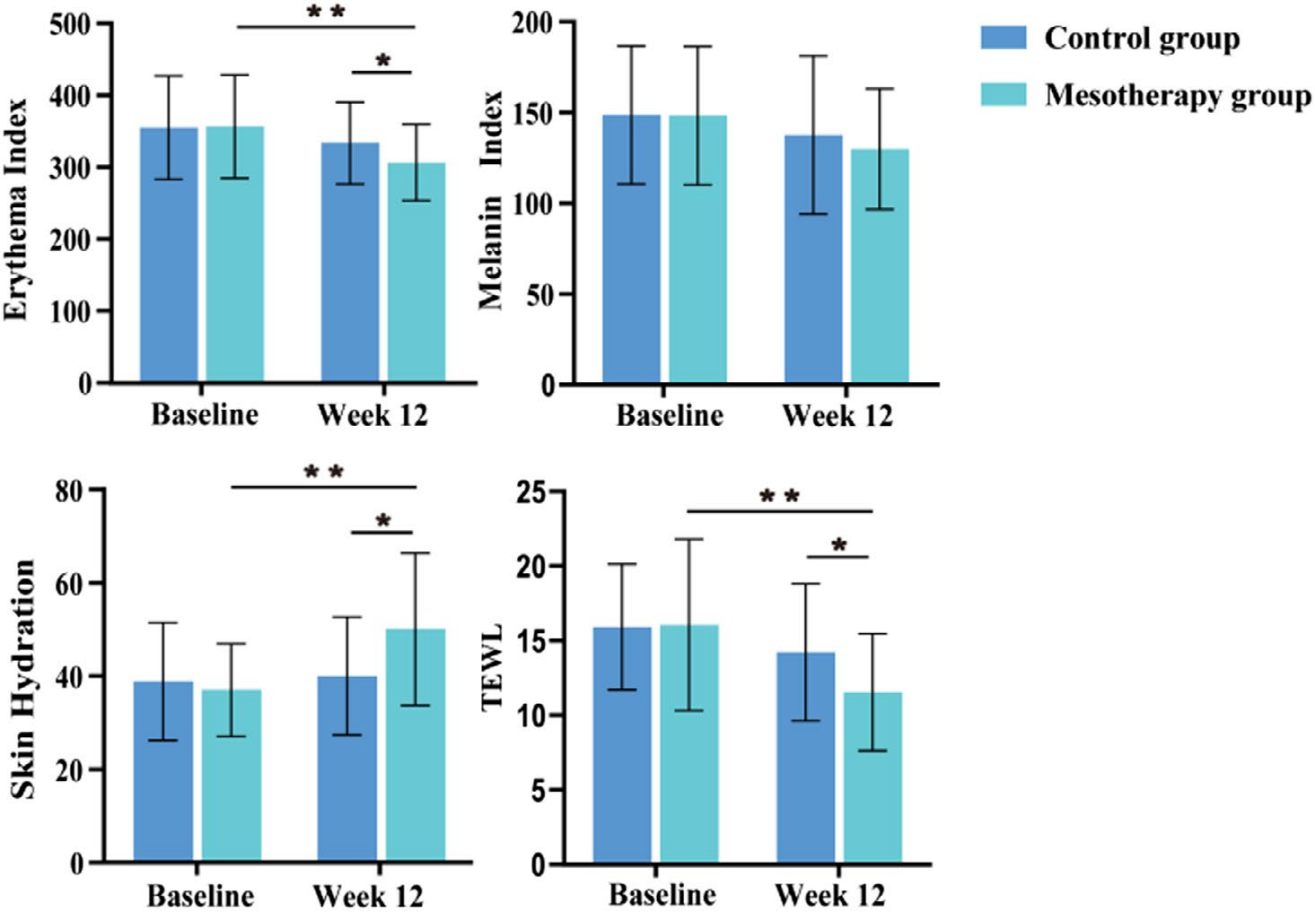
Comparison graphs of parameters measured by CK. *p < 0.05, **p < 0.01, ***p < 0.001.

There were no significant differences between the two groups in erythema index, melanin index, skin hydration, and TEWL at baseline (*p* > 0.05). The mesotherapy group showed significant improvements in erythema index, skin hydration, and TEWL compared to the control group, with a significant difference between the two groups at week 12 (all *p* < 0.05, Table 5 and Figure 5). Although both groups exhibited reductions in melanin index, these changes were not statistically significant either within or between groups.

**TABLE 5:** Comparison of parameters measured by CK.

	Baseline	Week 12	*p*
Erythema index			
Control group	355 ± 72	334 ± 57	0.217
Mesotherapy group	356 ± 72	307 ± 53	0.002*
*p*	0.950	0.041*	
Melanin index			
Control group	149 ± 38	138 ± 44	0.101
Mesotherapy group	148 ± 38	130 ± 33	0.079
*p*	0.953	0.805	
Skin hydration			
Control group	38.9 ± 12.6	40.0 ± 12.6	0.722
Mesotherapy group	37.0 ± 10.0	50.1 ± 16.3	0.006*
*p*	0.586	0.024*	
Transepidermal water loss			
Control group	15.9 ± 4.2	14.2 ± 4.6	0.285
Mesotherapy group	16.1 ± 5.7	11.6 ± 3.9	0.008*
*p*	0.926	0.039*	

* *p* < 0.05.

#### 3.2.5 Dermoscopic Imaging

**FIGURE 6:**
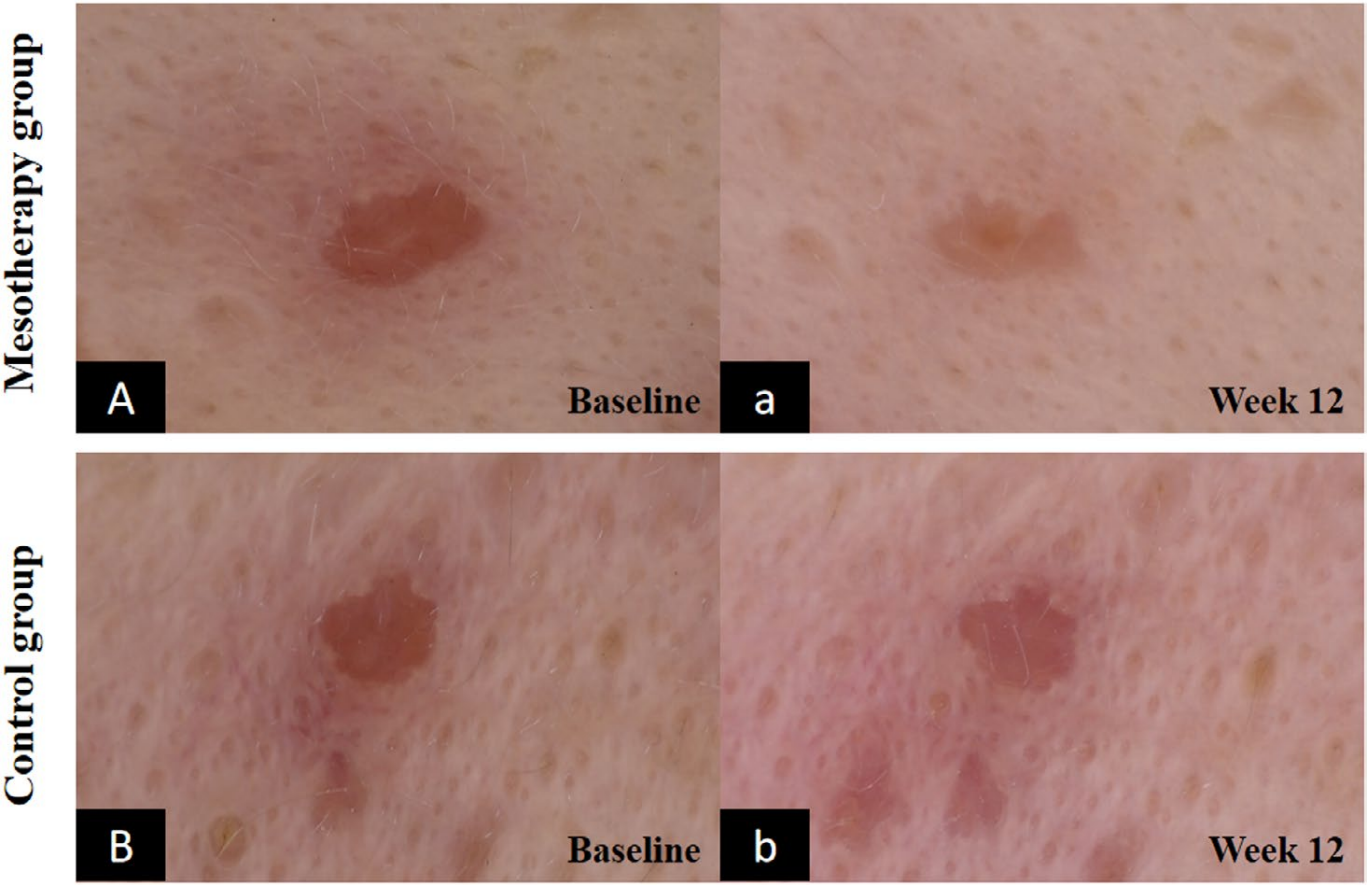
Dermoscopic imaging (40×) of a specific lesion at baseline and week 12. (A, B) Baseline. (a, b) Week 12. A, a: Mesotherapy group. B, b: Control group.

As shown in Figure 6, the mesotherapy group exhibited more marked improvements than the control group in the same facial lesion areas. This was evidenced by significantly greater reductions in erythema intensity, lesion size, and the severity of telangiectasia.

### 3.3 Adverse Effects

The procedures were well tolerated by all patients, with no serious adverse events reported. All patients experienced mild diffuse facial erythema associated with the treatment, which typically resolved within 1–3 days.

### 3.4 Relapse

Among the 23 patients who completed treatment, only one experienced acne relapse at week 24, with more lesions observed in the control group.

## 4 Discussion

According to our results, patients who received mesotherapy showed greater improvement in erythema, better patient self‐assessments, as well as marked enhancement of skin texture, hydration, and barrier function, compared to the control group. The results of this randomized split‐face trial suggest that non‐cross‐linked HA mesotherapy has a favorable safety and efficacy profile when used to treat PAE.

At week 12, the CEAS, red areas, and erythema index scores of the mesotherapy group were significantly lower than those of the control group, and dermoscopic imaging also demonstrated more pronounced clinical improvement. The main component of the mesotherapy used was non‐cross‐linked HA, which improves skin hydration and resilience through water‐holding capacity and barrier function repair [17], thereby reducing inflammation‐induced erythema and roughness. In addition, HA can bind to TLR receptors to regulate the release of pro‐inflammatory cytokines, exerting anti‐inflammatory effects [15] and it can also stimulate fibroblast proliferation and collagen synthesis, supporting dermal remodeling [18]. Consistent with our findings, Proietti et al. [19] reported that the combined injection of low‐molecular‐weight HA (LMW‐HA) for patients with rosacea, combined with local topical treatment, can improve skin texture and reduce the area of erythema. Additionally, longer needles were used in erythematous areas during mesotherapy to induce pinpoint bleeding and platelet aggregation. Platelets are rich in various growth factors (GFs), including transforming growth factor‐β1 (TGF‐β1), basic fibroblast growth factor (bFGF), platelet‐derived growth factor (PDGF), vascular endothelial growth factor (VEGF), and epidermal growth factor (EGF) [20]. These GFs promote treatment efficacy through various mechanisms, such as modulating inflammatory responses [21], promoting vascular remodeling, and facilitating dermal repair [20].

In terms of skin texture, the mesotherapy group showed significant improvements in texture and pores. Non‐cross‐linked HA mesotherapy delivers HA directly into the dermis to increase skin hydration. It also supports fibroblast function, thereby promoting collagen synthesis and enhancing overall skin quality [22]. Similarly, Wei et al. [23] demonstrated that intradermal injection of LMW‐HA significantly improved skin texture, reduced pores, and enhanced radiance. In addition, the mesotherapy formulation used in this study contained other functional components such as L‐carnosine, amino acids, and various vitamins. L‐carnosine has strong antioxidant activity, which can protect collagen fibers from oxidative damage and promotes collagen remodeling and wound healing [24]. Amino acids are essential substrates for fibroblast collagen synthesis [25], and vitamins are involved in collagen maturation and dermal stability [26]. These components may further enhance the improvement effect on skin texture through a synergistic effect.

The melanin index showed a decrease in both groups at week 12, but there was no statistically significant difference between the two groups. This may be because non‐cross‐linked HA itself does not contain active ingredients that inhibit melanin production or promote melanin metabolism, therefore lacking a direct whitening effect. The decrease of this index may be related to the improvement of the patient's skin moisture condition and strict sun protection.

The mesotherapy group showed a significant reduction in TEWL and a marked increase in skin hydration. TEWL is a key indicator for evaluating skin barrier integrity, with lower values reflecting better function. HA binds water molecules and enhances stratum corneum hydration, thereby reducing water loss and improving skin barrier function [14]. Laurino et al. [27] also reported that injections of a mixture of low‐molecular‐weight and high‐molecular‐weight HA could significantly increase skin hydration and reduce TEWL.

During the study period, no serious adverse events occurred in any of the patients. After the injections, all patients experienced mild, diffuse facial erythema, but these reactions were transient and usually resolved spontaneously within 1 to 3 days. Patients in the mesotherapy group reported higher satisfaction, and their DLQI scores were significantly lower compared to baseline. These results indicated that the non‐cross‐linked HA mesotherapy has high safety and tolerability.

Given the anti‐inflammatory properties of HA, we assessed acne relapse at week 24 to evaluate the long‐term impact of mesotherapy. Among the 23 participants who completed the study, only one experienced a relapse of acne, and the untreated control group showed more obvious symptoms. It might be related to the patient's chronic sleep deprivation. These findings further suggest that HA not only effectively controls inflammation but also provides long‐lasting therapeutic benefits.

The main advantage of this study was its split‐face design, which allowed an objective assessment of the effects of mesotherapy compared to the control group. It also effectively controlled for inter‐individual variability. To our knowledge, this is the first study specifically evaluating non‐cross‐linked HA for the treatment of PAE. This work expands the clinical use of a widely used compound and provides a potential alternative therapeutic approach to laser, lights, and topical agents.

This study also has several limitations. The sample size was relatively small, which may limit the generalizability of the findings. In addition, the follow‐up period was short, making it difficult to fully evaluate the long‐term efficacy and relapse of this treatment.

## 5 Conclusion

In conclusion, this trial demonstrated that non‐cross‐linked HA mesotherapy is a safe and effective treatment for post‐acne erythema. Compared to the control group, the mesotherapy group showed significant improvements in erythema severity, skin texture, hydration, and barrier function. Patients also reported higher patient satisfaction, and improved quality of life. The treatment was well tolerated, with only mild and transient adverse effects.

## Author Contributions

All authors contributed to the study conception and design. Material preparation, data collection and analysis were performed by Yuan Chang, Di Zhang, Yue Bai, Ansheng Tan, Zixuan He, Yu Han, Anxin Chen. The first draft of the manuscript was written by Yuan Chang. Fenglin Zhuo revised the manuscript. All authors read and approved the final manuscript.

## Funding

This work was supported by the Beijing Natural Science Foundation (L234068) and the National Natural Science Foundation of China (No. 82273555).

## Ethics Statement

2023‐8‐16, Ethics Review Committee of Beijing Friendship Hospital (2023‐P2‐209‐02).

## Consent

Prior to enrollment, patients had already agreed to the use of their photos and had signed the informed consent.

## Conflicts of Interest

The authors declare no conflicts of interest.

## Data Availability

The data that support the findings of this study are available on request from the corresponding author. The data are not publicly available due to privacy or ethical restrictions.
